# Aberrant DNA hydroxymethylation reshapes transcription factor binding in myeloid neoplasms

**DOI:** 10.1186/s13148-022-01297-5

**Published:** 2022-06-28

**Authors:** Jia Li, Tingting Hong, Yue Wei, Lei Guo, Minjung Lee, Hui Yang, Caleb Class, Yaling Yang, Xiaoqiong Wang, Hua He, Stefan Siwko, M. James You, Yubin Zhou, Guillermo Garcia-Manero, Yun Huang

**Affiliations:** 1 Center for Epigenetics and Disease Prevention, Institute of Biosciences and Technology, Texas A&M University, Room 404, 2121. W. Holcombe Blvd, Houston, TX 77030 USA; 2 Department of Leukemia, The University of Texas MD Anderson Cancer Center, Houston, TX 77030 USA; 3 Center for Translational Cancer Research, Institute of Biosciences and Technology, Texas A&M University, Houston, TX 77030 USA; 4 Department of Biostatistics, The University of Texas MD Anderson Cancer Center, Houston, USA

**Keywords:** DNA hydroxymethylation, Transcription factor, TET2, Myeloid neoplasms

## Abstract

Epigenetic abnormalities in DNA hydroxymethylation (5hmC) have been detected in patients with myeloid neoplasms, suggesting that 5hmC might act as a valuable epigenetic mark to reflect the disease status of myeloid neoplasms. Here, we report systematic genome-wide mapping of the DNA hydroxymethylomes in over 70 patients with myeloid neoplasms. Our integrative analysis leads to the identification of distinct 5hmC signatures that can sensitively discriminate patients from healthy individuals. At the molecular level, we unveiled dynamic 5hmC changes within key transcription factor (e.g., the CEBP family) binding motifs that are essential for hematopoiesis and myeloid lineage specification. 5hmC redistribution was found to alter the genome-wide binding of CEBP-α, thereby reprogramming transcriptional outputs to affect leukemia cell survival and stemness. Taken together, we provide a comprehensive 5hmC atlas representative of myeloid neoplasms, which sets the stage for future exploration on the epigenetic etiology of hematological malignancies. Mechanistically, our study further furnishes important insights into how abnormal 5hmC distribution in patients directly interrupts the binding of transcription factors to reshape transcriptional landscapes and aggravate leukemogenesis.

**Supplementary Information:**

The online version contains supplementary material available at 10.1186/s13148-022-01297-5.

## Introduction

Abnormal DNA hydroxymethylation has been reported in a myriad of human diseases, including solid tumors, hematological malignancies, and neurological disorders [1–4]. 5-hydroxymethylcytosine (5hmC) in the genome is an oxidation product of 5-methylcytosine (5mC) mediated by the TET protein family of epigenetic enzymes [5]. In normal cells, 5hmC is enriched at euchromatin regions with a relatively higher level at enhancers and coding regions compared with other genomic regions [6–10]. The enrichment of 5hmC at enhancers and coding regions is usually positively correlated with transcriptional activity. TET proteins are well known as tumor suppressors [11, 12]. The expression levels of TET enzymes are generally reduced in solid tumors, such as breast cancer and prostate cancer [13–15]. Somatic TET2 mutations are frequently reported in individuals with clonal hematopoiesis and various hematopoietic malignancies [16–18]. TET2 loss-of-function, arising from altered expression and/or somatic mutations, could lead to growth advantage and myeloid bias in hematopoietic stem and progenitor cells (HSPCs) [1, 19–21], which increases the risk of malignant transformation. Other factors could also indirectly influence TET activity and remodel the DNA hydroxymethylation landscape. For example, WT1 mutations have been shown to alter the chromatin association of TET2 [22]; while IDH1/2 mutations are found to impair the catalytic activity of TET2 via the production of 2-hydroxyglutarate [23], an oncometabolite that competes with the normal cofactor 2-oxoglutarate to alter enzymatic function with subsequent 5mC and 5hmC redistribution in HSPCs. Together, abnormal DNA hydroxymethylation arising from TET2 inactivation is regarded as one of the critical factors contributing to abnormal hematopoiesis and leukemogenesis. At the molecular level, 5hmC not only serves as an intermediate of DNA demethylation, but also actively functions as an important epigenetic mark to regulate 3D genome architecture [24, 25], chromatin accessibility [7, 26] and DNA damage response [27, 28]. Therefore, DNA hydroxymethylome could be exploited to reflect the disease status and facilitate the classification of molecular subtypes of hematological disorders and malignancies.

Transcription factors (TFs) are one of the fundamental components to orchestrate transcriptional outputs [29]. Cytosine modifications in DNA could directly modulate sequence-specific binding of TFs to the mammalian genome to alter downstream transcriptional activity [29, 30]. For example, proteins of the methyl CpG-binding domain (MBD) family recognize fully methylated CpG sequences and lead to transcriptional silencing [31]. UHRF2 as a 5hmC reader prefers to bind to 5hmC-containing DNA sequences and facilities active DNA demethylation [27, 32]. On the other hand, DNA cytosine modifications also can reduce TF binding. For instance, compared to unmodified cytosine-containing DNA, the MYC-binding partner MAX (Myc-associated factor X) engages 5mC- or 5hmC-containing oligos with > 400-fold reduction in the binding affinity [33]. We and others have also reported that DNA hydroxymethylation in the C/EBPꞵ binding motif substantially weakens the C/EBPꞵ-DNA interaction [34, 35]. These findings converge to support that abnormal 5hmC modification might lead to TFs redistribution to alter the transcriptional landscapes.

In this study, we applied a substantially improved genome-wide 5hmC profiling method [7, 10, 36] that is suitable for low-input DNA (as low as 10 ng) to obtain DNA hydroxymethylomes in more than 70 individuals with various myeloid neoplasms, including myelodysplastic syndrome (MDS), acute myeloid leukemia (AML) and chronic myelomonocytic leukemia (CMML). We observed distinct 5hmC patterns in patients with myeloid neoplasms compared with healthy controls, firmly establishing its diagnostic and prognostic values. In addition, we unveiled differential enrichment of 5hmC at various key transcription factor (TF) binding sites in genomic DNA samples isolated from bone marrows of these patients. Using the C/EBP family as an example, we revealed a strong negative correlation between 5hmC enrichment and C/EBP binding in human leukemia cells, which provides direct evidence to demonstrate the correlation between DNA hydroxymethylation and TF binding. Overall, our study not only yields a first-of-its-kind comprehensive genome-wide 5hmC atlas representative of myeloid neoplasms, but also provides a solid foundation to better interrogate the epigenetic etiology of cancer.

## Results

### 5hmC landscapes in patients with myeloid neoplasms

**Fig. 1:**
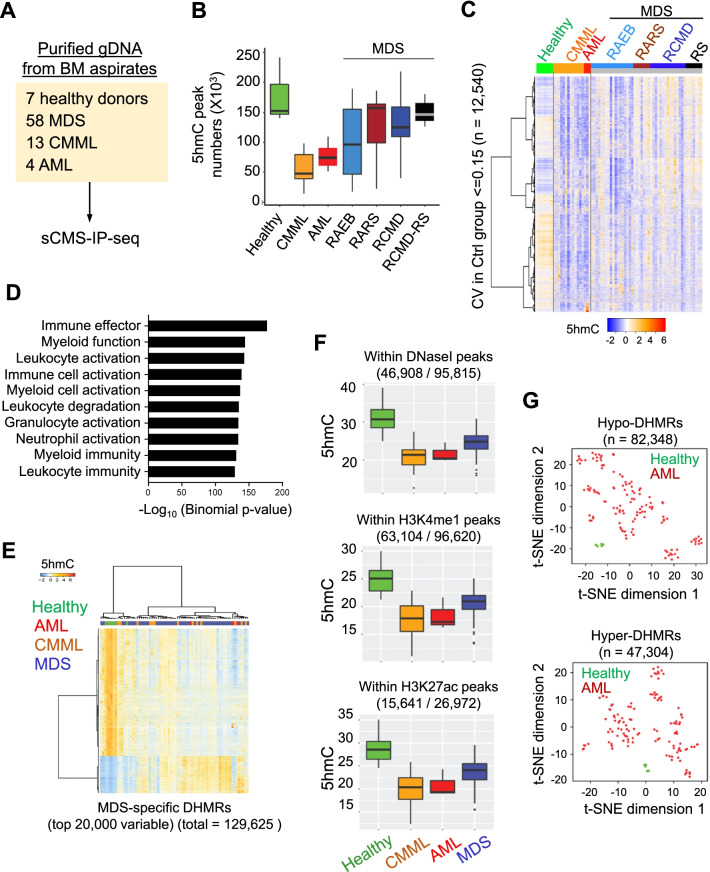
Heterogenous 5hmC distributions in patients with myeloid neoplasms. **A** The experimental design for genome-wide sCMS-IP-seq analysis in the cohort. **B** Boxplot representation of the identified 5hmC peak numbers in the analyzed cohort. Bounds of the box span from 25 to 75% percentile, and the center line within each box represents the median. Whiskers represent median ± 1.5 times interquartile range. **C** Heatmap representation of the enrichment of selected 5hmC peaks among all the analyzed cohorts. The selected 5hmC peaks (*n* = 12,540) exhibited small variations (coefficient of variation, CV < 0.15) in healthy donor groups, whereas MDS and cancer patients showed higher variation. Each row represents a selected 5hmC peak, each column represents an individual person. RAEB: Refractory anemia with excess blasts; RARS: Refractory anemia with ring sideroblasts; RCMD: Refractory cytopenia with multilineage dysplasia; RS: Ring sideroblasts. **D** Top 10 selected categories of the GREAT analysis results for the 5hmC peaks shown in Fig. 1C. **E** Heatmap representation of the top 20,000 variable disease-specific differentially hydroxymethylated regions (DHMRs). **F** Box-plot showing the enrichment of 5hmC within published DNaseI hypersensitive sites, H3K4me1-, or H3K27ac-enriched genomic regions in healthy controls, as well as patients diagnosed with myeloid neoplasms. Bounds of the box span from 25 to 75% percentile, the center line within the box represents the median. Whiskers represent median ± 1.5 times interquartile range. **G** The t-SNE plot of the DNA methylation level within disease-specific DHMRs in the published AML cohort. Hypo-DHMRs: genomic regions showed decreased 5hmC levels in patients compared with healthy controls; Hyper-DHMRs: genomic regions showed increased 5hmC levels in patients compared with healthy controls. The DNA methylation levels at each CpG site were obtained from published datasets collected from CD34 + cells in healthy control (phs000159) or bone marrow aspirates in AML patients (GSE98350)

We performed 5hmC analysis in a total of 82 bone marrow aspirates from 75 patients with myeloid neoplasms and 7 healthy controls (Additional file 1: Table S1, Fig. 1A). The mutation status of these patients was listed in Additional file 1: Fig. S1. In order to profile genome-wide 5hmC with clinical samples, we greatly improved our previously published CMS-IP-seq method [7, 10, 36, 37] and made it fully compatible with low-input genomic DNA (as low as 10 ng; Additional file 1: Fig. S2A-D, Fig. 1). We collected 1.05 billion uniquely mapped reads (1,021,637,350 reads) that covered 7.2% of the human genome with an average length of 5hmC peaks at ~ 200 bp. By comparing the 5hmC peak numbers among analyzed individuals, we observed more 5hmC peaks in healthy donors (174,399 peaks on average) compared with patients diagnosed with chronic myelomonocytic leukemia (CMML; 68,625 peaks) and adult acute myeloid leukemia (AML; 77,371 peaks) (Additional file 1: Fig. S2E). MDS patients displayed the highest variations in 5hmC peak numbers, which ranged from 10,728 to 217,132 peaks (average 108,315 peaks) regardless of MDS subtypes (Fig. 1B). Among the different MDS subtypes, patients with refractory cytopenia with multilineage dysplasia and ringed sideroblasts (RCMD-RS) displayed similar numbers of 5hmC peaks as healthy controls; however, the genomic location of 5hmC peaks was distinct between RCMD-RS and healthy donors, suggesting a unique molecular feature of RCMD-RS (Fig. 1C). Besides the variable numbers of 5hmC peaks identified from MDS patients, we also observed higher heterogeneity of the genomic 5hmC distributions in MDS patients compared with healthy donors, AML and CMML patients, further echoing the heterogenous features of MDS patients observed in the clinic [38] (Fig. 1C, Additional file 1: Fig. S2E-F). Genomic Regions Enrichment of Annotations Tool (GREAT) analysis further revealed that the genomic regions displaying high 5hmC heterogeneity in MDS patients (*n* = 12,540) were enriched at genes essential for hematopoietic and immune cell function [39] (Fig. 1D).

Despite of the heterogeneity of 5hmC distributions among analyzed patients, we were still able to identify 129,652 conserved disease-specific 5hmC peaks (differentially hydroxymethylated regions, DHMRs) (Fig. 1E). GREAT analysis [39] indicated that these DHMRs were associated with genes that are relevant for myeloid and lymphoid cell function (Additional file 1: Fig. S3A). To further examine the distribution of 5hmC with other epigenetic marks in patients with myeloid neoplasms, we analyzed the 5hmC enrichment profile within the published DNase I hypersensitive site and histone modification (H3K4me1 and H3K27ac) regions collected from human CD34 + cells (Fig. 1F, Additional file 1: Table S2). We observed significantly reduced 5hmC levels in DNase I hypersensitive sites, H3K4me1, and H3K27ac enriched regions, suggesting impaired DNA hydroxymethylation at enhancer regions in patients with MDS and myeloid malignancies (Fig. 1F). In parallel, we analyzed DNA methylation levels within the DHMRs using publicly available whole-genome bisulfite sequencing (WGBS) in healthy CD34 + hematopoietic stem progenitor cells (HSPCs) and reduced representation bisulfite sequencing (RRBS) data collected from AML patients [40] (Fig. 1G and Additional file 1: Fig. S3B, Table S2). We observed a high similarity of DNA methylation level in CpG sites located within DHMRs in healthy donors. By contrast, the same CpG sites in AML patients had high DNA methylation variations (Fig. 1G and Additional file 1: Fig. S3B). Collectively, these data suggest epigenetic abnormalities, including altered DNA methylation, histone modifications and chromatin accessibility at DHMRs, represent a hallmark of myeloid neoplasms.

### Distinct 5hmC features in patients with myeloid neoplasms

**Fig. 2:**
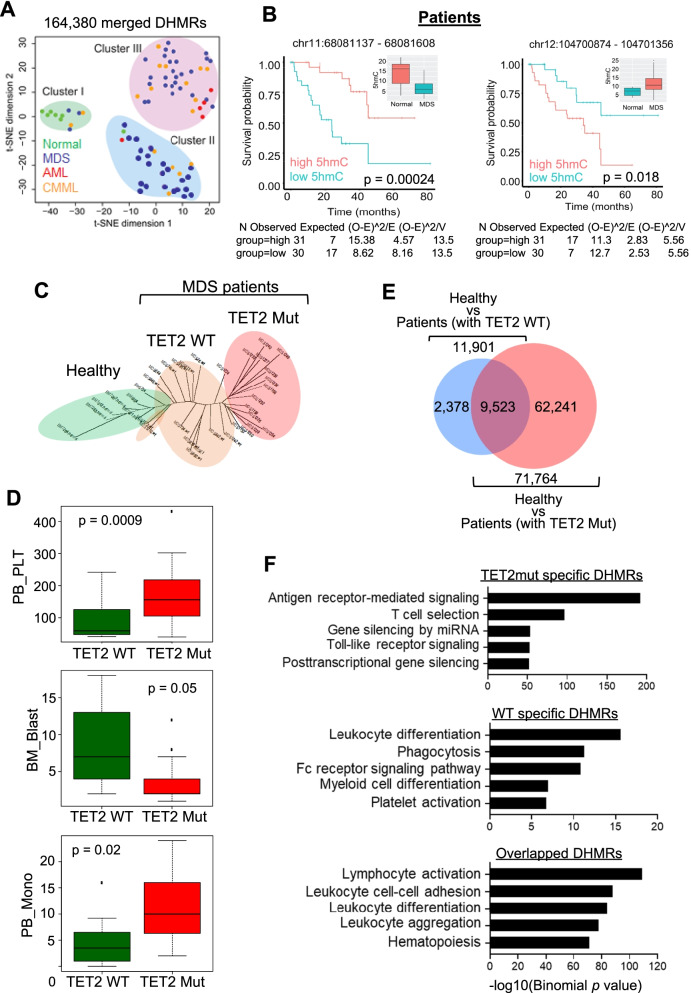
The distinct clustering patterns of disease-specific DHMRs. **A** The t-SNE plot for the merged DHMRs identified from the analyzed cohort. Three distinct clusters were observed, reflecting distinct disease status. **B** Kaplan–Meier survival curves for patients with high and low 5hmC levels at selected genomic regions. The high and low 5hmC were separated by the median value of 5hmC. Boxplot: Bounds of the box span from 25 to 75% percentile, the center line within each box represents the median. Whiskers represent median ± 1.5 times interquartile range. **C** The clustering analysis of DHMRs based on TET2 mutation status in healthy controls and patients with myeloid neoplasms (with WT or mutant TET2). **D** The immunophenotypic features, including platelet (PLT), blasts, and monocytes (mono) counting in patients with WT TET2 and mutant TET2. **E** Venn diagram of DHMRs identified between the comparison of healthy donors vs patients with WT TET2 or healthy donors vs patients with mutant TET2. **F** GREAT analysis of DHMRs identified from the comparison shown in (**E**)

To further evaluate the clinic implication of landscape changes in 5hmC, we performed t-Distributed Stochastic Neighbor Embedding (t-SNE) analysis using the 5hmC profiles within the tested cohort (Fig. 2A). Interestingly, the healthy controls clustered far from with patients bearing myeloid neoplasms (Cluster I vs Clusters II & III; Fig. 2A). In addition, we used all identified DHMRs to correlate 5hmC alterations with the survival of analyzed patients. 622 DHMRs showed a strong association (*P* < 0.05) with survival (Additional file 1: Table S3), which could be used as potential biomarkers to predict patient outcomes in future clinical management, as example regions shown in Fig. 2B and Additional file 1: Fig. S4A. GREAT analysis [39] revealed that these survival-associated DHMRs were correlated with genes that regulate lymphocyte activation and differentiation (Additional file 1: Fig. S4B).

DNA hydroxymethylation is catalyzed by the TET protein family member, with TET2 being one of the most frequently mutated genes in patients with myeloid neoplasms. Therefore, we further investigated whether we could combine 5hmC and TET2 mutation information to facilitate the molecular classification of the disease. Among all the analyzed patients, 30 patients had annotated TET2 mutation status (12 with WT TET2 and 18 with mutant TET2). We then compared the 5hmC distributions within healthy controls and patients with either WT or mutant TET2. As shown in Fig. 2C, we were able to clearly separate these three groups of patients based on the differential 5hmC landscapes and TET2 mutation status. Patients with TET2 mutations exhibited more notable differences than those with WT TET2 when both groups were compared to the healthy controls (Fig. 2C). Next, we further examined the clinical features of patients with and without TET2 mutations (Fig. 2D). We observed that individuals with TET2 mutations had higher platelet and monocyte counts but lower blast counts compared with those bearing normal TET2 (Fig. 2D). No significant differences in neutrophil numbers, white blood counts and hemoglobin levels were noted between these two groups (Additional file 1: Fig. S4C). These results agreed with previous reports from us and others showing that TET2 mutations tend to cause myeloid bias in both human and mouse studies [18–20].

To further understand the impact of TET2 on 5hmC landscapes and downstream transcriptional regulation, we compared 5hmC profiles among normal healthy donors, patients with and without TET2 mutations based on the mutation information obtained from Additional file 1: Fig. S1 and Table S1. Compared to the healthy donor group, we identified a total of 11,910 and 71,764 differentially hydroxymethylated region (DHMRs) in patients with WT TET2 or with TET2 mutations, respectively. Among these DHMRs, 9,523 were found to be independent on TET2 mutation status (Fig. 2E). In parallel, we identified 2,378 and 62,241 DHMRs that were specifically enriched in patients with WT or mutant TET2, respectively (Fig. 2E). The GREAT analysis showed that all the DHMRs identified from Fig. 2E were enriched at genomic regions that are related to immune cell activation, suggesting an abnormal inflammation feature in myeloid cancer patients regardless of TET2 mutation status (Fig. 2F). We also observed that the DHMRs specifically identified in TET2-mutant patients were enriched at genomic regions associated with genes important for T cell function and post-transcriptional silencing (Fig. 2F), suggesting that TET2 mutations have strong impact on reshaping the 5hmC landscapes and altering transcriptional activities in patients with myeloid neoplasms.

### Abnormal 5hmC enrichment at key transcription factor (TF) binding sites in myeloid neoplasms

**Fig. 3:**
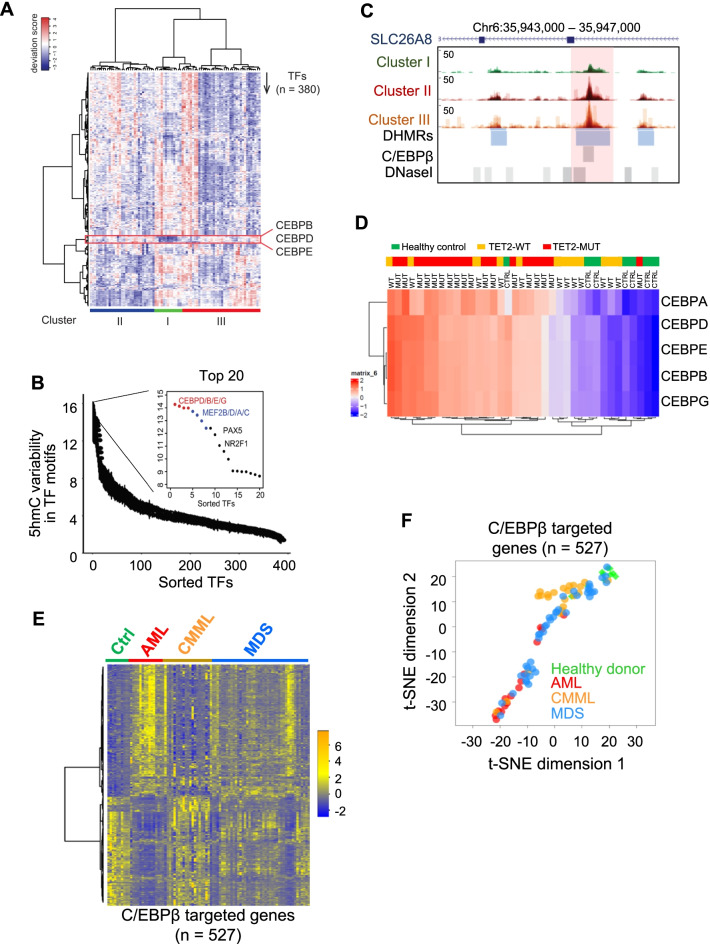
Abnormal 5hmC enrichment at key TF binding sites in patients with myeloid neoplasms. **A** Heatmap representation of the 5hmC deviation score at the annotated TF-binding motifs (*n* = 380). Higher deviation scores represent more enrichment of 5hmC in corresponding TFs motifs; negative deviation scores mean depletion of 5hmC in TF-binding motifs. Each row represents an individual TF motif. Each column stands for an individual case. Red box, 5hmC enrichment status of the indicated C/EBP family members. **B** The rank of 5hmC changes within the analyzed TF-binding motifs. The C/EBP family members are generally ranked among the top 10 mostly-enriched motifs. **C** Genome-browser views of the overlaid 5hmC enrichment at the C/EBPβ binding sites. The 5hmC signals within each individual at each cluster were overlaid. C/EBPβ binding sites were obtained from the public C/EBPβ ChIP-seq datasets (GSM2345026 and GSM2345027). **D** Heatmap representation of 5hmC deviation scores in healthy donors and patients with known TET2 mutation status (WT vs mutation) at the binding motifs of the C/EBP families. **E** Heatmap representation of the expression of C/EBPβ target genes [46] (*n* = 527) in the analyzed cohort. The C/EBPβ target genes were defined as genes containing C/EBPβ binding sites within 1-kb of their transcription start site. The C/EBPβ binding sites were identified from public ChIP-seq data (GSM2345026 and GSM2345027). **F** The t-SNE analysis on the expression of C/EBPβ target genes [46] in healthy donors and patients with AML, CMML or MDS

Our current study showed significant 5hmC alterations within DNase I hypersensitive sites marked by enhancer marks, H3K4me1 and H3K27ac, in subjects with myeloid neoplasm compared to healthy individuals (Fig. 1F). Previous studies have shown that 5hmC is enriched at enhancers occupied by transcription factors (TFs) [7, 9], and that DNA hydroxymethylation could alter TF binding [34, 41]. We therefore examined 5hmC enrichment within 380 TF motifs in the analyzed cohort using a previously developed analysis pipeline [42]. Indeed, we observed substantial 5hmC changes within these TF motifs (Fig. 3A). Overall, 5hmC levels within these TF motifs were high in healthy individuals (mostly in Cluster I) but remained low in patients (mostly in Cluster II and Cluster III). Interestingly, among all the analyzed motifs, we observed a set of TF motifs, belonging to the CCAAT-enhancer-binding protein (C/EBP) family, that displayed a distinct 5hmC distribution pattern (Fig. 3A-C), with low 5hmC levels in healthy individuals (Cluster I) but high 5hmC in patients (Clusters II and III). In addition, patients with TET2 mutations exhibited similar enrichment of 5hmC within C/EBP binding sites compared with patients with WT TET2 or healthy controls (Fig. 3D, Additional file 1: Fig. S5A). Furthermore, this set of C/EBP binding motifs displayed the most significant changes in 5hmC between healthy individuals and patients (comparison between Cluster I and Cluster II & III) (Fig. 3B-C). C/EBP family members have been reported to play an essential role in regulating hematopoiesis and myeloid differentiation [43, 44]. Genetic defects in C/EBP have been reported in AML [45]. Furthermore, our previous study demonstrated that 5hmC modification could prevent C/EBPβ DNA binding *in vitro* [34], suggesting that increased 5hmC within C/EBP binding motifs might reshape genomic location of C/EBP and its downstream transcriptional activity during leukemogenesis. Indeed, consistent with changes in DNA hydroxymethylation, the expression levels of C/EBPβ target genes [46] were significantly altered in patients with myeloid neoplasms (Fig. 3E-F).

To seek other potential TFs with 5hmC changes within their binding motifs in samples from myeloid neoplasms, we extended our analyses to other TFs and correlated 5hmC levels within all 380 TF motifs with the overall survival of patients. Differential 5hmC levels within 24 additional TFs were detected and were correlated with patient outcomes (Additional file 1: Fig. S5B). For example, distinct 5hmC levels within TP63 and MYBL2 binding regions were significantly associated with the survival of patients with myeloid neoplasms (Additional file 1: Fig. S5C). Overall, these data establish that patients with myeloid neoplasms display dramatic 5hmC changes within key TF binding motifs and that 5hmC changes within selected TF binding motifs are strongly associated with patient outcomes.

### Aberrant 5hmC enrichment reshapes CEBP-α binding in human leukemia cells

**Fig. 4:**
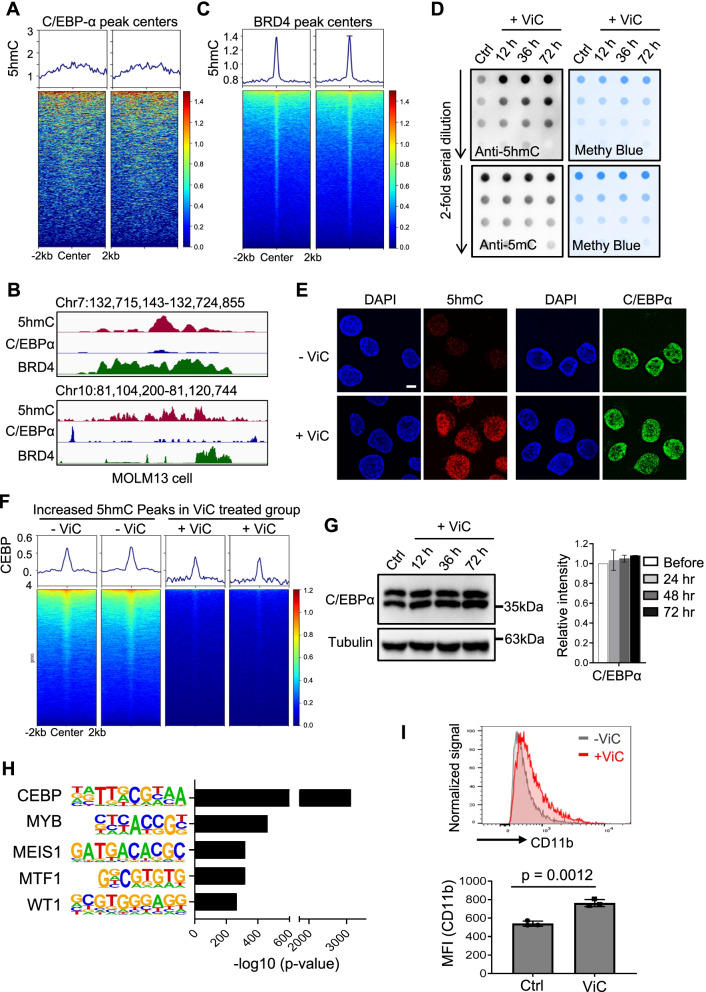
5hmC enrichment reshapes CEBP-α binding in human leukemia cells. **A** Histogram and Heatmap representation of 5hmC enrichment within the C/EBP-α binding sites in MOLM13 leukemia cells. **B** Genome-browser views of 5hmC (red), C/EBP-α (blue) and BRD4 (green) peaks in MOLM13 cells at the indicated regions. BRD4, but not C/EBP-α, was enriched at 5hmC-enriched regions. **C** Histogram and Heatmap representation of 5hmC enrichment within the BRD4 binding sites in MOLM13 cells. BRD4 ChIP-seq data were obtained from GSM1557123. A total of 54,411 BRD4 peaks were identified. **D** Dotblot analysis of global 5hmC (top) and 5mC (bottom) levels in MOLM13 cells treated with or without 250 µM Vitamin C (ViC) at the indicated time points. Methylene blue (Methyl Blue) staining was used on the same blot as the loading control. ViC treatment led to 5hmC increase, but had minor effects on 5mC levels in MOLM13 cells. **E** Immunofluorescent staining of 5hmC (red) and C/EBPα (green) in MOLM13 cells before and after Vitamin C (ViC) treatment for 72 h. DAPI was used for nuclear staining. ViC treatment resulted in a significant increase of 5hmC, but had minor effects on the fluorescent signal of C/EBPα. Scale bar: 5 µm. **F** Histogram and Heatmap representation of C/EBP-α enrichment within the newly emerged 5hmC peaks following ViC treatment in MOLM13 cells compared with the untreated group. C/EBP-α enrichment was plotted within the new 5hmC peaks gained in ViC-treated MOLM13 cells. C/EBP-α enrichment was significantly reduced in regions showing ViC-induced 5hmC increase. **G** Immunoblot (left) and statistical quantification (right) of C/EBP-α protein expression in MOLM13 cells treated without and with 250 µM ViC at the indicated time points. Anti-tubulin was used as the loading control. *n* = 3 biological replicates. **H** Motif analysis of DHMRs identified in MOLM13 cells before and after ViC (250 µM) treatment for 72 h. **I** Representative histogram and statistical analysis of flow cytometry analysis on CD11b expression in MOLM13 cells treated with or without ViC (250 µM) for 72 h. MFI: mean fluorescent intensity. Data were shown as mean ± S.D; *n* = 3. *P* = 0.0012, by two-tailed Student’s t-test

To further evaluate the impact of 5hmC on C/EBP binding in the disease context of leukemia, we performed genome-wide 5hmC and C/EBP analysis in MOLM13, a human AML cell line. Because C/EBP-α, but not C/EBP-β, is highly expressed in MOLM13 cell (Additional file 1: Fig. S6A) and genetic lesions in C/EBP-α contribute to AML development [45], we used C/EBP-α as a proof-of-concept example to investigate the causal relationship between 5hmC and C/EBP-α enrichment in MOLM13 cell. To achieve this, we performed C/EBP-α ChIP-seq and sCMS-IP-seq in MOLM13 cell. We observed almost no enrichment of 5hmC within the C/EBP-α binding sites (Fig. 4A-B, Additional file 1: Fig. S6B). To ensure the data quality of 5hmC profiling, we also monitored the 5hmC enrichment within BRD4 binding sites [47] in MOLM13 cells because 5hmC is known to be enriched at enhancers and BRD4 is an enhancer binding protein. We observed strong 5hmC enrichment within the BRD4 binding sties (Fig. 4B-C), which ruled out the possibility that the absence of 5hmC enrichment at C/EBP-α binding sites is due to low sCMS-IP-seq data quality. To further investigate whether 5hmC indeed repels genomic binding of C/EBP-α, we treated MOLM13 cells with vitamin C (ViC), which is known to boost the TET enzymatic activity to increase global 5hmC levels without altering the expression levels of *TET* family members (Fig. 4D-E, Additional file 1: Fig. S6C-D). ChIP-seq analysis confirmed that vitamin C induced 5hmC enhancement significantly and suppressed genomic association of C/EBP-α without altering its protein expression level and its chromatin association capability (Fig. 4E-G, Additional file 1: Fig. S6E-F). Motif analysis also confirmed that C/EBP binding motif was enriched most prominently in genomic regions that  exhibited  ViC-induced gain of DNA hydroxymethylation (Fig. 4H). These results strongly indicate that ViC-mediated increase in 5hmC reshapes the genomic location of C/EBP-α. In addition, GREAT analysis implied that genomic regions with enhanced 5hmC are associated with genes essential for cell survival and stress response (Additional file 1: Fig. S6G). ViC-treated MOLM13 cells have been previously shown to upregulate the expression of genes involved in apoptotic and cell differentiation signaling [48]. Likewise, in our functional analysis, we also observed upregulation of CD11b expression in MOLM13 cells following ViC treatment (Fig. 4I), suggesting that 5hmC might induce re-localization of C/EBP-α to promote differentiation of AML cells. Overall, our data strongly suggest a direct effect of 5hmC in regulating genomic distribution of C/EBP-α in leukemia cells. TET2 loss of function could alter 5hmC distribution and reshape C/EBP-α binding to impact downstream transcriptional activities during leukemogenesis.

## Discussion

In this study, we reported the use of a substantially improved, highly sensitive CMS-IP-seq (sCMS-IP-Seq) method [10, 36] for genome-wide 5hmC profiling using low-input DNA samples from patients. This method can be widely applied to rapidly map 5hmC landscapes in rare clinical samples. With this method, we analyzed genome-wide 5hmC distributions in bone marrow DNA samples derived from over 80 healthy donors and patients with myeloid neoplasms or immunotherapy, including MDS, AML and CMML. We observed dynamic 5hmC alterations in patients with myeloid neoplasms compared with healthy donors. In particular, the 5hmC distributions within MDS patients displayed high heterogeneity when compared with the patterns in AML and CMML patients. Since the analysis was performed with mixed bone marrow aspirates, it is unclear whether the observed 5hmC heterogeneity stems from the mixed cell populations or because of intrinsic molecular variations within the same cell types. Therefore, single-cell epigenome profiling technology seems to be imperative to clarify between these two possibilities in near-future follow-on studies.

Although we observed high variations of 5hmC enrichment within the analyzed cohort, 5hmC landscape still can be used to clearly separate healthy donors and patients with myeloid neoplasms. In addition, patients with TET2 mutations exhibited distinct 5hmC landscapes compared with those bearing WT TET2. These findings point to the potential high prognostic value of 5hmC and TET2 in classifying patients with myeloid neoplasms. Within all the genome-wide 5hmC alterations, we observed dramatic changes of 5hmC at TF binding motifs in patients with myeloid neoplasms. The enrichment of 5hmC at TF binding sites has been reported in previous studies [7, 9, 26, 49], which could either directly modulate TF binding to DNA [34] or alter the accessibility of chromatin where TF binds [24]. Alterations of 5hmC at TF binding sites could further impinge on myeloid specific transcription and lead to myeloid neoplasms. In the current study, 5hmC changes at these TFs are significantly associated with the overall survival in our analyzed cohort of patients. We also observed a subset of differentially expressed genes that are closely correlated with 5hmC changes at specific TF-binding motifs, as exemplified by the C/EBP family members. In most analyzed TF binding regions, we observed decreased 5hmC in patients with myeloid neoplasms compared with healthy donors. However, we observed significantly increased 5hmC levels within the C/EBP family binding motifs. Our previous in vitro biochemical studies suggested that 5hmC modification could reduce C/EBP binding to its genomic targets [34, 35]. In this study, using genome-wide profiling analysis, we further confirmed mutual exclusion between genomic enrichment of 5hmC and C/EBP genomic binding in leukemia cells. The functional consequence of such interplay is to reprogram the transcriptional outputs that ultimately might contribute to leukemogenesis. However, it remains puzzling why 5hmC is specifically enriched in the C/EBP binding sites in patients with myeloid neoplasms regardless of a global decrease in 5hmC distribution within other TF binding motifs. Further experiments and analysis in purified progenitor cells are required to resolve this interesting question.

Although the transcriptional level of C/EBP remains unchanged in all the analyzed samples, including cancer cell lines, we also observed transcriptional alterations of various TFs in human bone marrow aspirates in this study (Additional file 1: Fig. S7). Many of those affected TFs are reported to regulate hematopoiesis and myeloid functions. The alteration of their gene expression and 5hmC changes within their binding motifs might synergize together to promote pathogenesis of myeloid neoplasm. Therefore, further ChIP-seq and ATAC-seq [50] analysis in these samples could be carried out to define the correlation between 5hmC and TF binding during myeloid cancer development.

In summary, we have employed a novel sCMS-IP-seq method to systematically generate a comprehensive DNA hydroxymethylome atals using limited DNA samples collected from bone marrow aspirates of over 70 patients with myeloid neoplasms. With these large-scale datasets, we have identified distinct epigenetic lesions reflecting the clinical features and outcomes of patients. At the molecular level, we identified differential 5hmC enrichment at selected TF binding motifs, e.g., C/EBP protein family, which reshapes their genomic location and reprograms transcriptional outputs to aggravate myeloid tumorigenesis. Collectively, our findings suggest the importance of 5hmC in myeloid neoplasms disease progression and establish 5hmC as a potential biomarker for the clinical management of patients with myeloid neoplasms.

## Materials and methods

### Bone marrow sample collection and gDNA purification

Bone marrow (BM) aspirates were obtained from patients via needle aspiration. The diagnosis of MDS, CMML or AML was confirmed by a hematopathologist. BM samples from healthy individuals were obtained from AllCells (Emeryville, CA). BM mononuclear cells were isolated using a Ficoll-Paque density gradient media (GE, Sweden). Genomic DNA (gDNA) was extracted using the DNeasy Blood & Tissue Kit (Qiagen).

### Highly sensitive CMS-IP sequencing (sCMS-IP-seq)

sCMS-IP-seq was performed as described previously [36] with some modifications. Briefly, genomic DNA was combined with unmethylated λ-bacteriophage DNA (NEB) for the bisulfite conversion efficiency check and then sheared into a median 300-bp fragment size range using a Covaris M220 Focused-ultrasonicator (Covaris). Sheared DNA was bisulfite converted using an EZ DNA methylation-lightning kit (Zymo Research) with suggested protocols to convert 5-hydroxymethylcytosine (5hmC) to cytosine-5-methylenesulfonate (CMS). CMS-containing DNA fragments were then immunoprecipitated using a CMS-specific antiserum pre-conjugated with protein A/G dynabeads. Precipitated DNA fragments were purified by the conventional phenol/ chloroform/ isoamyl-alcohol method. Purified enriched fragments were amplified with random primers followed by adaptor ligation. Final amplification was performed with illumina TruSeq indices using a Pico Methyl-Seq Library Prep Kit (Zymo Research) by following the manufacturer’s protocols. Constructed library size distribution was determined by a Bioanalyzer with an Agilent High Sensitivity DNA Kit (Agilent). Library concentration was measured by a Qubit 4 Fluorometer using a Qubit dsDNA high sensitivity assay kit (Thermo Fisher Scientific). Pooled DNA libraries were sequenced on NextSeq 500 (Illumina) with a NextSeq 500/550 High Output Kit v2 (single-end reads, 75 cycles) following protocols suggested by the manufacturer (Illumina).

### sCMS-IP-seq data analysis

The analysis pipeline is available at https://github.com/lijinbio/HaMiP. Raw fastq data for sCMS-IP were aligned to hg19 using BSMAP [51]. After discarding PCR duplicated reads, only the uniquely mapped reads were kept for downstream normalization and further analyses. We further removed duplicated reads and used the unique reads to identify enriched peak regions for each sample using MACS2 [52] with default parameters to call CMS-IP-seq peaks. A count table including the raw counts for each peak across all the samples was generated using an in-house script. DESeq2 [53] was used to normalize the reads count for each sample and to identify the differentially hydroxymethylated (CMS-IP) peaks (DHMRs; FDR <  = 0.05, when compared with health donor samples). To facilitate the visualization of hydroxymethylation (5hmC) signals, bigWig files for read coverage were generated from the aligned BAM files and visualized in the UCSC genome browser. Functional annotations of DHMRs were assigned using the GREAT analysis with default settings [39]. The R package ggplot2 was used to plot violin plots and boxplots. Heatmap with hierarchical clustering was generated using the R package heatmap3 (https://www.rdocumentation.org/packages/heatmap3/versions/1.1.6/ topics/heatmap3). t-Distributed Stochastic Neighbor Embedding (t-SNE) analysis was performed using the R package Rtsne (https://github.com/jkrijthe/Rtsne). The smoothed scatterplots were plotted using the R package: geneplotter.

### Integrative analysis of 5hmC within TF-binding motifs

The R package chromVAR (https://github.com/GreenleafLab/chromVAR) was used to analyze 5hmC signal changes in TF binding regions across all patient samples. The DHMRs, the aligned bam file of CMS-IP-seq data and TF motif position weight matrices (PWMs) were used as inputs. First, chromVAR calculated the ‘raw deviation’, the difference between the total numbers of fragments that map to regions/peaks containing the motif and the expected numbers of fragments. The raw deviations for background peak sets were used to compute a bias-corrected deviation and z-score for each annotation and sample. This provided a differential measure of the gain or loss of 5hmC for a given genomic annotation relative to the average sample profile. The Motif analysis of DHMRs were performed using the HOMER motif analysis package.

### Correlation analysis between 5hmC and overall survival (OS)

We selected pre-treatment samples to perform this analysis. OS was defined as the time from the patient diagnosis to disease related death or the last follow-up. Next, we performed multivariate Cox regression analysis using 5hmC signals from identified DHMRs. The 5hmC signatures of regions significantly associated with OS (*P* < 0.05) were defined as potential prognostic markers. The “survival” and “survminer” functions in the R package were used to visualize the survival curves.

### MOLM13 cell culture and Vitamin C treatment

MOLM13 cells were obtained from ATCC and maintained in RPMI1640 (Corning, cat#: 10–040-CV) with 10% FBS (Omega Scientific, Ca#: FB-11) and 1% Penicillin–Streptomycin (Sigma, Cat#: P4333) under 5% CO2 at 37ºC. Vitamin C was purchased from Sigma (Cat#: A7631) and dissolved in PBS. MOLM13 cells were treated with 250 µM Vitamin C for the indicated duration and an equal amount of PBS was used as control.

### Dot-blot analysis of global 5hmC levels

DNA was purified using Takara NucleoSpin Tissue kit and denatured in 0.4 M NaOH, 10 mM EDTA at 95 °C for 10 min, then neutralized with ice-cold 2 M ammonium acetate (pH 7.0). Two-fold serial dilutions of the denatured DNA samples were generated and spotted on a nitrocellulose membrane by using an assembled Bio-Dot apparatus (Bio-Rad) according to the manufacturer’s instructions. The membrane was washed with 2xSSC buffer briefly, air-dried and vacuum-baked at 80 °C for 2 h. DNA hybridized membrane was blocked with 5% non-fat milk for 1 h at room temperature and incubated with an anti-5hmC antibody (1:3000, Active Motif, Cat# 39,769) overnight at 4 °C. Next day, the membrane was incubated with a horseradish peroxidase-conjugated anti-rabbit IgG secondary antibody (1:3000, Cell Signaling, cat# 7074S) for 1 h at room temperature. The membrane was visualized by West-Q Pico Dura ECL Solution (GenDEPOT). The membrane was washed with 1X TBST briefly and then stained with 0.02% methylene blue in 0.3 M sodium acetate (pH 5.2) to confirm the total amounts of loaded DNA samples.

### Western blot and chromatin fraction assays

For total protein, MOLM13 cell pellets were lysed in a RIPA buffer and loaded to 4% to 12% gradient SDS-PAGE (GenScript) gels by mixing with SDS loading buffer (100 mM pH6.8 Tris–Cl, 4% SDS, 0.2% bromophenol blue, 20% glycerol, 200 mM DTT) after denaturation at 95 °C for 10 min. For chromatin fraction assay, MOLM13 cells were lysed in buffer A (10 mM HEPES, pH 7.9, 10 mM KCl, 1.5 mM MgCl_2_, 0.34 M sucrose, 10% glycerol, 0.1% Triton X-100, 1 mM DTT, and protease inhibitor cocktails) to remove the cytoplasm. Nuclear pellets were enriched by centrifugation at 1,300 g at 4 °C for 5 min, followed by Buffer N treatment (15 mM Tris–HCl [pH 7.5], 200 mM NaCl, 60 mM KCl, 5 mM MgCl_2_, 1 mM CaCl_2_, 0.3% NP-40, and protease inhibitor cocktails) for 30 min. After centrifugation at 1,700 g, 4 ˚C, for 5 min, the supernatant was removed, and 100 µl sample loading buffer was added to the chromatin pellets for denaturing as the chromatin binding fraction. Denatured proteins were loaded to the 4% to 12% gradient SDS-PAGE (GenScript). Nitrocellulose membranes (Millipore) for western blot analysis. Anti-C/EBP-α (1:1000, Santa Cruz, cat# sc-166258) and anti-C/EBP-β (1:1000, Santa Cruz, cat# sc-7962), and anti-tubulin (dilution, vendor, cat#) antibodies were used as primary antibodies. HRP conjugated anti-mouse IgG (1:3000, Cell signaling, cat# 7076S) and HRP conjugated anti-Rabbit IgG (1:3000, Cell Signaling, cat# 7074S) were used as secondary antibodies.

### CEBP-α ChIP-seq analysis

10 million MOML13 cells were crosslinked with 1% v/v methanol-free formaldehyde at room temperature for 10 min with gentle rotation. Crosslinking was quenched by adding glycine to a final concentration 0.125 M and incubated at room temperature for another 5 min with gentle rotation. Cells pellets were collected by centrifugation at 500 g for 5 min and washed twice with cold PBS containing protease inhibitor cocktail. Cell pellets can be stored at -80 °C for several months after discarding the supernatant. Next, 15 μl protein G and 15 μl protein A magnetic beads (per IP sample) were washed twice with 1xRIPA buffer (10 mM Tris pH 7.5, 1 mM EDTA, 1% Triton X-100, 0.1% SDS, 0.1% sodium deoxycholate, 100 mM NaCl and freshly added proteinase inhibitor cocktail). Beads were resuspended with 500 μl 1xRIPA buffer and 10 μg CEBPα antibody (Santa Cruz, cat# sc-166258), followed by rotating at 4 °C for at least 3 h. Pellets from 2 million crosslinked cells were resuspended with 130 μl 0.25% SDS sonication buffer (10 mM Tris–HCl pH 8.0, 0.25% SDS, 2 mM EDTA) and sonicated by Covaris for 10 min according to the manufacturers’ instructions. The sonicated lysates were further diluted with 1.5-fold of an equilibration buffer (10 mM Tris,233 mM NaCl, 1.66%TritonX-100, 0.166% DOX, 1 mM EDTA, and proteinase inhibitor cocktail). The combined cell lysates (from 10 million cells) were centrifuged at 12,000 rpm, 4 °C for 10 min to pellet the insoluble fraction. Supernatants (with 1/10 taken out as input) were transferred to a new tube and combined with antibody conjugated magnetic beads, and incubated at 4 °C overnight with gentle rotation. Next day, beads were washed twice with low salt RIPA buffer, high salt RIPA buffer, LiCl buffer and TE buffer. For reverse crosslinking, beads were resuspended with 300 μl elution buffer (20 mM Tri-HCl, pH7.5, 5 mM EDTA, 50 mM NaCl, 1% SDS, 50 μg/ml proteinase K) and incubated at a thermomixer at 68 °C, 1100 rpm for at least 3 h. The eluted chromatins were extracted with phenol:chloroform (with 1:1 ratio) and further precipitated by cold ethanol. Input DNA and Immunoprecipitated DNA were quantified by Qubit and then proceeded to library preparations using a Takara ThruPLEX DNA-seq kit (R400674) according to the manufacturer’s instructions. Library concentrations were measured by Qubit and library sizes were analyzed by Bioanalyzer (Agilent Genomics). Multiplexing indexed libraries were pooled and quantified by Kapa library quantification kit and sequenced on an Illumina Nextseq 500 platform.

## Supplementary Information

**Additional file 1**. Supplementary information.

## Data Availability

The sequencing datasets have been deposited into NCBI BioProject under the accession number GSE148357.
