# Supplementary material for: Aberrant DNA hydroxymethylation reshapes transcription factor binding in myeloid neoplasms
Source: Clin Epigenetics. 2022 Jun 28;14:81. doi: 10.1186/s13148-022-01297-5 (PMC9241241; doi:10.1186/s13148-022-01297-5)
Supplement: Supplementary file 1 — Additional file 1. Supplementary information. [file 13148_2022_1297_MOESM1_ESM.pdf]

Supplementary information

Aberrant DNA hydroxymethylation alters transcription factor binding to reshape transcriptional landscapes in myeloid neoplasms

Jia Li[1*#], Tingting Hong[1*], Yue Wei[2], Lei Guo[1], Minjung Lee[1], Hui Yang[2], Caleb Class[2], Yaling Yang[2], Xiaoqiong Wang[2], Hua He[2], Stefan Siwko[3], M. James You[2], Yubin Zhou[3], Guillermo Garcia-Manero[2,#], Yun Huang[1#]

1. Center for Epigenetics & Disease Prevention, Institute of Biosciences and Technology Texas A&M University, Houston, TX 77030
2. Department of Leukemia, The University of Texas MD Anderson Cancer Center Houston, TX 77030
3. Center for Translational Cancer Research, Institute of Biosciences and Technology Texas A&M University, Houston, TX 77030

* Equal contribution

# Correspondence: jiali@tamu.edu; ggarciam@mdanderson.org; yun.huang@tamu.edu

Lead contact:

Yun Huang, PhD
Room 404, 2121. W. Holcombe Blvd
Houston, TX 77030
yun.huang@tamu.edu, 713-677-7484

Materials and Methods

Integrative analysis of 5hmC within TF-binding motifs

The R package chromVAR (https://github.com/GreenleafLab/chromVAR) was used to analyze 5hmC signal changes in TF binding regions across all patient samples. The DHMRs, the aligned bam file of CMS-IP-seq data and TF motif position weight matrices (PWMs) were used as inputs. First, chromVAR calculated the ‘raw deviation’, the difference between the total numbers of fragments that map to regions/peaks containing the motif and the expected numbers of fragments. The raw deviations for background peak sets were used to compute a bias-corrected deviation and z-score for each annotation and sample. This provided a differential measure of the gain or loss of 5hmC for a given genomic annotation relative to the average sample profile. The Motif analysis of DHMRs were performed using the HOMER motif analysis package.

Correlation analysis between 5hmC and overall survival (OS)

We selected pre-treatment samples to perform this analysis. OS was defined as the time from the patient diagnosis to disease related death or the last follow-up. Next, we performed multivariate Cox regression analysis using 5hmC signals from identified DHMRs. The 5hmC signatures of regions significantly associated with OS ($p < 0.05$) were defined as potential prognostic markers. The “survival” and “survminer” functions in the R package were used to visualize the survival curves.

MOLM13 cell culture and Vitamin C treatment

MOLM13 cells were obtained from ATCC and maintained in RPMI1640 (Corning, cat#: 10-040-CV) with 10% FBS (Omega Scientific, Ca#: FB-11) and 1% Penicillin-Streptomycin (Sigma, Cat#: P4333) under 5% CO_2 at 37ºC. Vitamin C was purchased from Sigma (Cat#: A7631) and dissolved in PBS. MOLM13 cells were treated with 250 µM Vitamin C for the indicated duration and an equal amount of PBS was used as control.

Dot-blot analysis of global 5hmC levels

DNA was purified using Takara NucleoSpin Tissue kit and denatured in 0.4 M NaOH, 10 mM EDTA at 95 °C for 10 min, then neutralized with ice-cold 2 M ammonium acetate (pH 7.0). Two-fold serial dilutions of the denatured DNA samples were generated and spotted on a nitrocellulose membrane by using an assembled Bio-Dot apparatus (Bio-Rad) according to the manufacturer’s instructions. The membrane was washed with 2xSSC buffer briefly, air-dried and vacuum-baked at 80°C for 2 h. DNA hybridized membrane was blocked with 5% non-fat milk for 1 hour at room temperature and incubated with an anti-5hmC antibody (1:3000, Active Motif,

Cat# 39769) overnight at 4°C. Next day, the membrane was incubated with a horseradish peroxidase-conjugated anti-rabbit IgG secondary antibody (1:3000, Cell Signaling, cat# 7074S) for 1 hour at room temperature. The membrane was visualized by West-Q Pico Dura ECL Solution (GenDEPOT). The membrane was washed with 1X TBST briefly and then stained with 0.02% methylene blue in 0.3 M sodium acetate (pH 5.2) to confirm the total amounts of loaded DNA samples.

Western blot and chromatin fraction assays

For total protein, MOLM13 cell pellets were lysed in a RIPA buffer and loaded to 4% to 12% gradient SDS-PAGE (GenScript) gels by mixing with SDS loading buffer (100 mM pH6.8 Tris-Cl, 4% SDS, 0.2% bromophenol blue, 20% glycerol, 200 mM DTT) after denaturation at 95°C for 10 minutes. For chromatin fraction assay, MOLM13 cells were lysed in buffer A (10 mM HEPES, pH 7.9, 10 mM KCl, 1.5 mM $MgCl_2$, 0.34 M sucrose, 10 % glycerol, 0.1% Triton X-100, 1 mM DTT, and protease inhibitor cocktails) to remove the cytoplasm. Nuclear pellets were enriched by centrifugation at 1,300 g at 4 °C for 5 min, followed by Buffer N treatment (15 mM Tris-HCl [pH 7.5], 200 mM NaCl, 60 mM KCl, 5 mM $MgCl_2$, 1 mM $CaCl_2$, 0.3% NP-40, and protease inhibitor cocktails) for 30 min. After centrifugation at 1,700 g, 4 ˚C, for 5 min, the supernatant was removed, and 100 µl sample loading buffer was added to the chromatin pellets for denaturing as the chromatin binding fraction. Denatured proteins were loaded to the 4% to 12% gradient SDS-PAGE (GenScript). Nitrocellulose membranes (Millipore) for western blot analysis. Anti-C/EBP-α (1:1000, Santa Cruz, cat# sc-166258) and anti-C/EBP-β (1:1000, Santa Cruz, cat# sc-7962), and anti-tubulin (dilution, vendor, cat#) antibodies were used as primary antibodies. HRP conjugated anti-mouse IgG (1:3000, Cell signaling, cat# 7076S) and HRP conjugated anti-Rabbit IgG (1:3000, Cell Signaling, cat# 7074S) were used as secondary antibodies.

Immunofluorescence staining

MOLM13 cells were concentrated onto poly-D-lysine pre-coated microscope slides through Cytospin. The cells were fixed with freshly prepared 4% paraformaldehyde solution (PFA) for 15 min at room temperature. After washing three times with PBS, cells were permeabilized with 0.2% Triton X-100 in PBS. For 5hmC staining, cells were treated with 2N HCl for 30 mins followed by neutralization with 100 mM Tris-HCl (pH 8.5) for 10 mins and then proceeded to the blocking step. For C/EBP-α staining, cells were directly blocked with 1% BSA / 0.05% Tween-20 / PBS at room temperature for 1 hr without HCl treatment. Then cells were incubated with primary antibodies

(anti-5hmC or anti-C/EBPα) in a blocking buffer overnight at 4 °C. Cells were then washed three times with PBS and incubated with secondary antibodies in the blocking buffer at room temperature for 1 hr. Cells were then covered with antifade mountant (with DAPI) for further imaging. A W1 Yokogawa Ti2 Nikon spinning disk confocal microscope was used for imaging acquisition and analysis.

RNA isolation and real-time quantitative PCR (RT-qPCR)

Total RNA was extracted using an AllPrep DNA/RNA Micro Kit (Qiagen) by following the manufacturer's instructions. DNA was cleared through DNase I digestion. cDNA synthesis was achieved using a PrimeScript™ 1st strand cDNA Synthesis Kit (Takara). Gene expression was quantified on a ViiA 7 Real-Time PCR System (Applied Biosystems) using 2× Universal SYBR Green Fast qPCR Mix (ABclonal). Primers used for RT-qPCR were listed below:

TET1 For: GCAGCGTACAGGCCACCACT

TET1 Rev: AGCCGGTCGGCCATTGGAAG

TET2 For: TTCGCAGAAGCAGCAGTGAAGAG

TET2 Rev: AGCCAGAGACAGCGGGATTCCTT

TET3 For: GACGAGAACATCGGCGGCGT

TET3 Rev: GTGGCAGCGGTTGGGCTTCT

CEBP-α ChIP-seq analysis

10 million MOML13 cells were crosslinked with 1% v/v methanol-free formaldehyde at room temperature for 10 min with gentle rotation. Crosslinking was quenched by adding glycine to a final concentration 0.125M and incubated at room temperature for another 5 min with gentle rotation. Cells pellets were collected by centrifugation at 500 g for 5 min and washed twice with cold PBS containing protease inhibitor cocktail. Cell pellets can be stored at -80 °C for several months after discarding the supernatant. Next, 15 µl protein G and 15 µl protein A magnetic beads (per IP sample) were washed twice with 1xRIPA buffer (10mM Tris pH 7.5, 1mM EDTA, 1% Triton X-100, 0.1% SDS, 0.1% sodium deoxycholate, 100mM NaCl and freshly added proteinase inhibitor cocktail). Beads were resuspended with 500 µl 1xRIPA buffer and 10 µg CEBPα antibody (Santa Cruz, cat# sc-166258), followed by rotating at 4 °C for at least 3 hours. Pellets from 2 million crosslinked cells were resuspended with 130 µl 0.25% SDS sonication buffer (10 mM Tris-HCl pH 8.0, 0.25% SDS, 2mM EDTA) and sonicated by Covaris for 10 min according to the manufacturers' instructions. The sonicated lysates were further diluted with 1.5-fold of an equilibration buffer (10mM Tris, 233 mM

NaCl, 1.66 %TritonX-100, 0.166 % DOX, 1 mM EDTA, and proteinase inhibitor cocktail). The combined cell lysates (from 10 million cells) were centrifuged at 12,000 rpm, 4 °C for 10 min to pellet the insoluble fraction. Supernatants (with 1/10 taken out as input) were transferred to a new tube and combined with antibody conjugated magnetic beads, and incubated at 4 °C overnight with gentle rotation. Next day, beads were washed twice with low salt RIPA buffer, high salt RIPA buffer, LiCl buffer and TE buffer. For reverse crosslinking, beads were resuspended with 300 µl elution buffer (20mM Tri-HCl, pH7.5, 5mM EDTA, 50mM NaCl, 1% SDS, 50 ug/ml proteinase K) and incubated at a thermomixer at 68 °C, 1100 rpm for at least 3 hours. The eluted chromatins were extracted with phenol:chloroform (with 1:1 ratio) and further precipitated by cold ethanol. Input DNA and Immunoprecipitated DNA were quantified by Qubit and then proceeded to library preparations using a Takara ThruPLEX DNA-seq kit (R400674) according to the manufacturer’s instructions. Library concentrations were measured by Qubit and library sizes were analyzed by Bioanalyzer (Agilent Genomics). Multiplexing indexed libraries were pooled and quantified by Kapa library quantification kit and sequenced on an Illumina Nextseq 500 platform.

Extended Data Figures

Figure S1

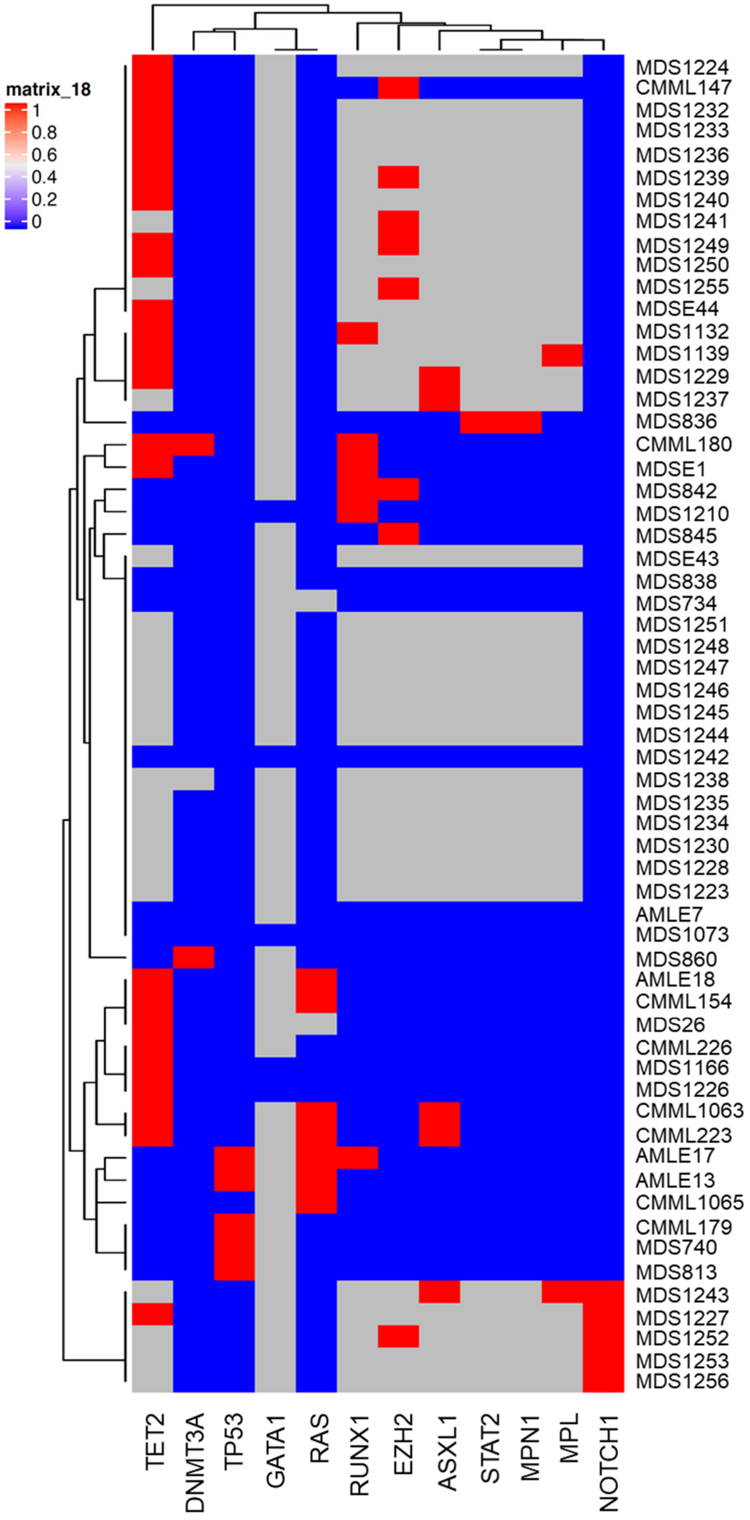

Figure S1. Mutation status annotation of the analyzed cohort.

Heatmap representation of mutation status of our analyzed patient cohort. Each row represents an individual patient, and each column represents one analyzed gene. Red indicates a mutation; Blue indicates WT; Grey means unknown.

Figure S2

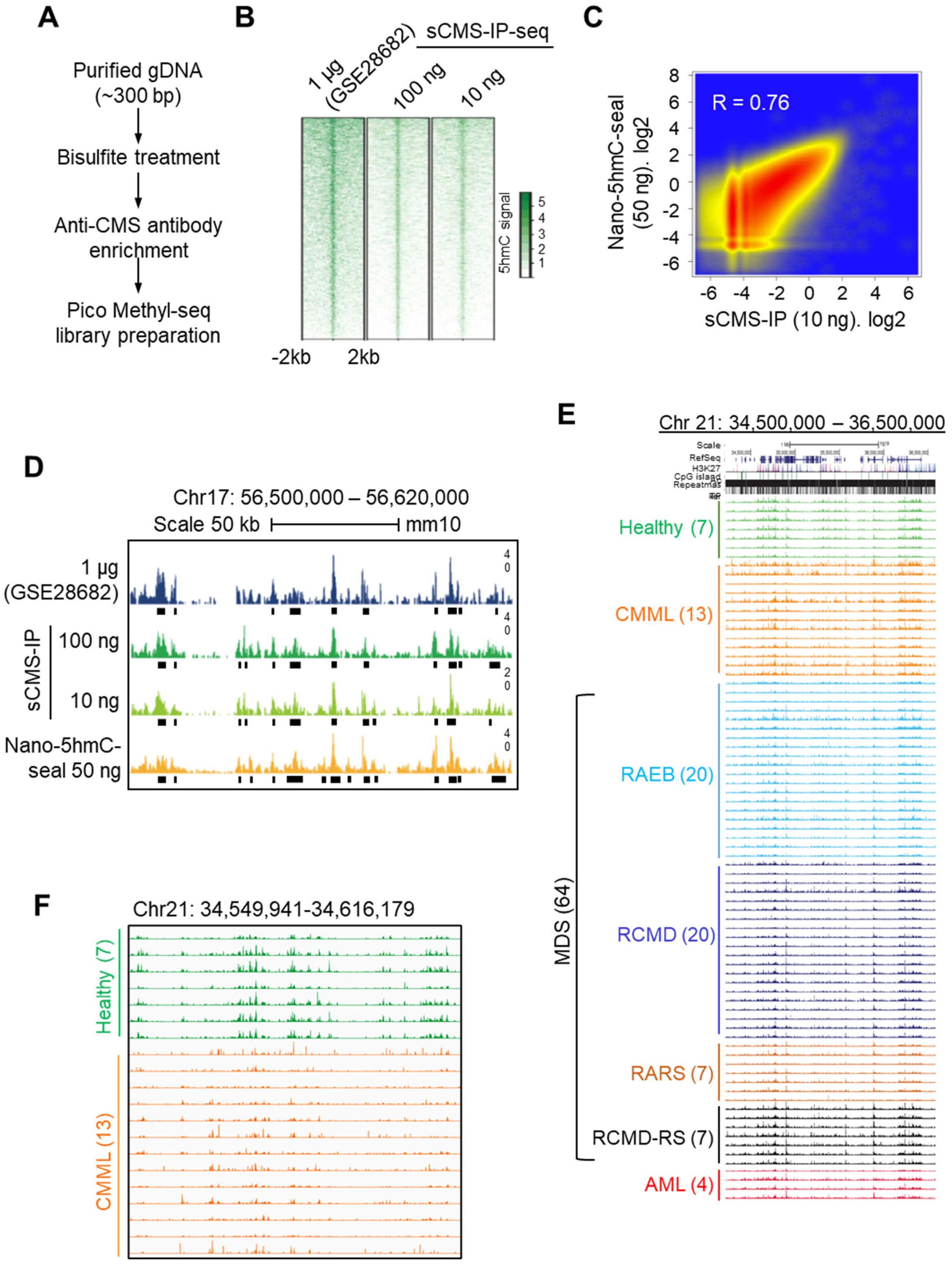
A
Purified gDNA (~300 bp)
Bisulfite treatment
Anti-CMS antibody enrichment
Pico Methyl-seq library preparation
B
sCMS-IP-seq
1 µg (GSE28682)
100 ng
10 ng
5hmC signal
-2kb
2kb
C
R = 0.76
Nano-5hmC-seal (50 ng). log2
sCMS-IP (10 ng). log2
D
Chr17: 56,500,000 – 56,620,000
Scale 50 kb
mm10
1 µg (GSE28682)
sCMS-IP
100 ng
10 ng
Nano-5hmC-seal 50 ng
E
Chr 21: 34,500,000 – 36,500,000
Healthy (7)
CMML (13)
MDS (64)
RAEB (20)
RCMD (20)
RARS (7)
RCMD-RS (7)
AML (4)
F
Chr21: 34,549,941-34,616,179
Healthy (7)
CMML (13)

Figure S2. sCMS-IP-seq for genome-wide profiling of DNA hydroxymethylome using low-input DNA.

(A) The work-flow of sCMS-IP-seq.

(B) Heatmap representation of 5-hmC profiling results using the regular CMS-IP-seq method (GSE28682) and the sCMS-IP-seq method (100 ng and 10 ng input). The gDNA input amounts for CMS-IP were indicated on the top of the heatmap. The up- and down- 2kb of the center of 5hmC peaks identified in regular CMS-IP-seq were used to plot the heatmaps.

(C) Pearson correlation analysis of genome-wide 5hmC analysis using the sCMS-IP-seq method (10 ng gDNA) and the published nano-5hmC-seal method (50 ng gDNA) (GSE77967) in mESCs. The Pearson correlation coefficient was determined as 0.76.

(D) Genome-browser views of 5hmC distribution in a genomic locus identified using the regular CMS-IP-seq method (GSE28682), the sCMS-IP-seq method (100 ng and 10 ng input) and the nano-5hmC-seal method (50 ng input) (GSE77967). The black bars below each track represent the identified peaks.

(E) Genome-browser views of 5hmC distributions of selected genomic regions in different patients.

(F) A zoom-in view of genome browser snapshots of 5hmC distribution in healthy donor and CMML patients.

Figure S3

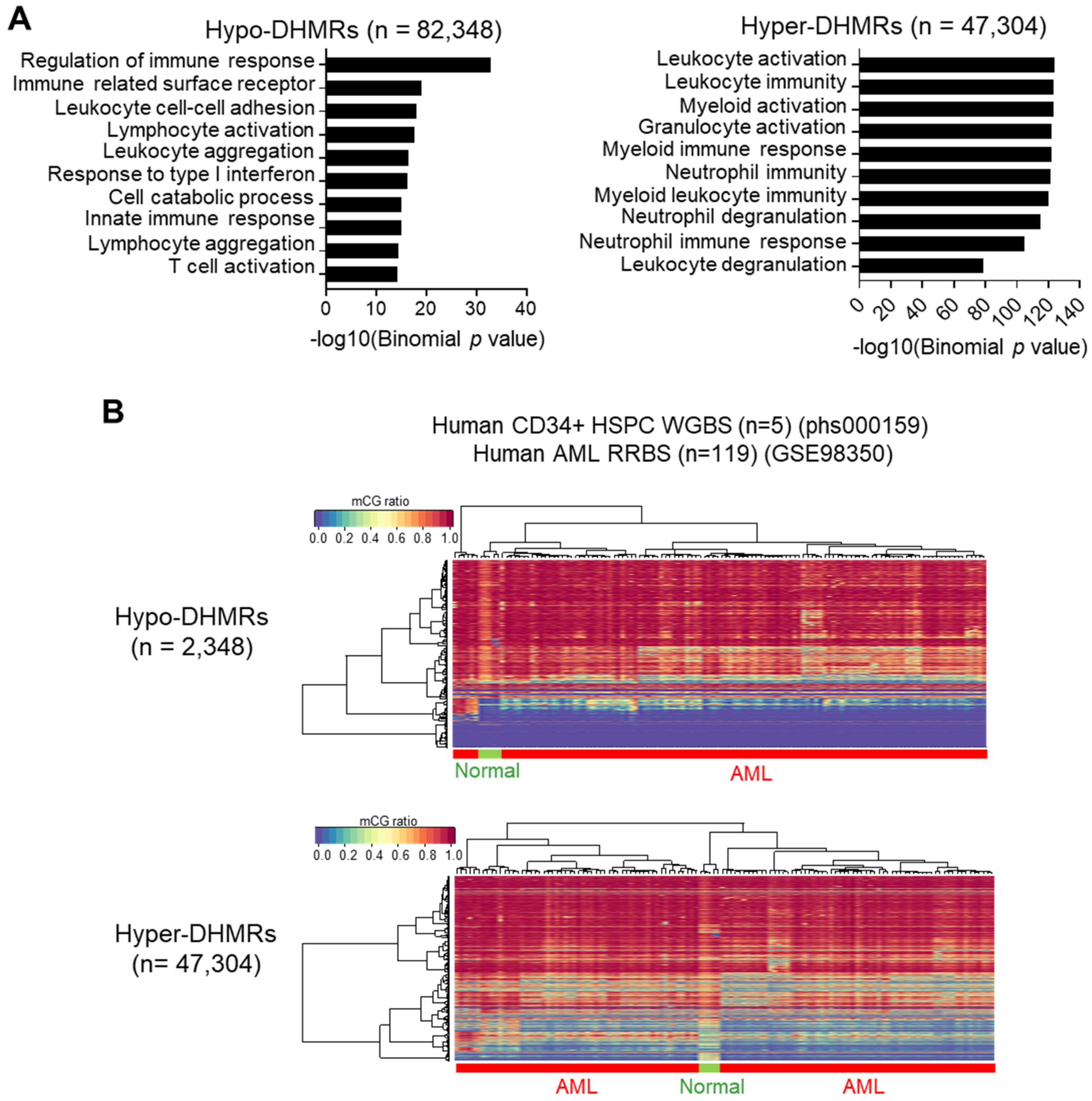

Figure S3. The characterization of disease-specific DHMRs.

(A) GREAT analysis of MDS-specific hypo-DHMRs and hyper-DHMRs.

(B) Heatmap representation of DNA methylation of CpG sites within MDS-specific hypo-DHMRs and hyper-DHMRs. The DNA methylation levels of individual CpG sites were obtained from published datasets collected from CD34+ cells in healthy controls (phs000159) or bone marrow aspirates in AML patients (GSE98350). Each row presents the DNA methylation level of individual CpGs; each column stands for an individual person.

Figure S4

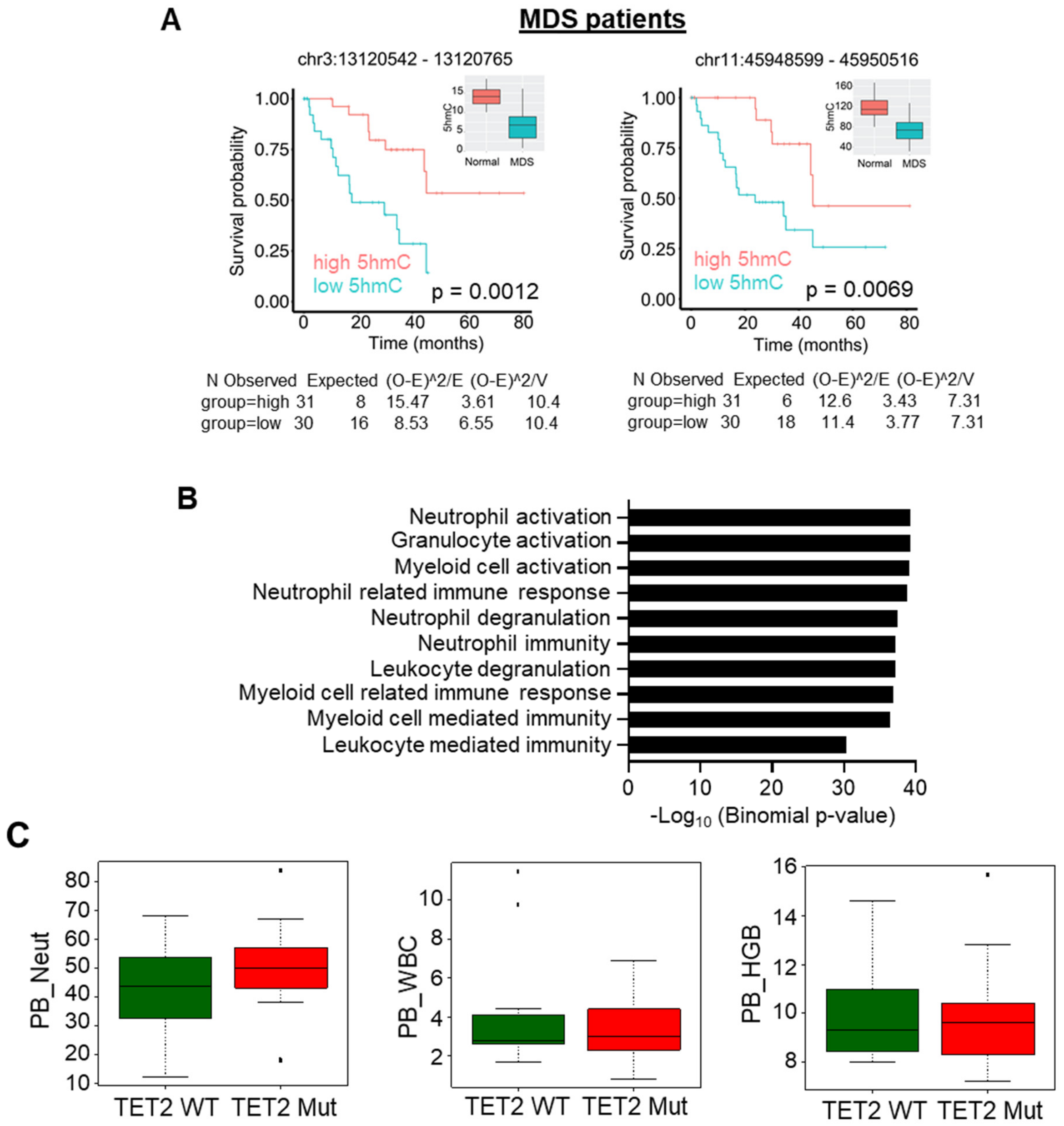

Figure S4. The clustering of disease-specific DHMRs.

(A) Kaplan-Meier survival curves for patients with high and low 5hmC levels at selected genomic regions. The high and low 5hmC groups were separated by the median value of 5hmC. Boxplot: Bounds of the box span from 25 to 75% percentile, the center line within each box represents the median. Whiskers represent median ± 1.5 times interquartile range. (B) GREAT analysis of DHMRs significantly associated with the overall survival of patients. (C) The clinical features, including hemoglobin (HGB), white blood cells (WBC) and neutrophil counts, among the analyzed patients with and without TET2 mutations.

Figure S5

A

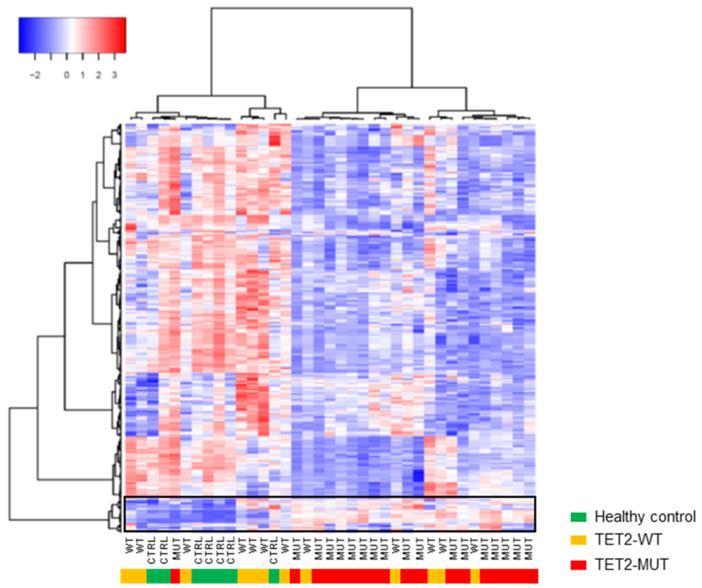

B

List of TFs motifs where 5hmC enrichment is correlated with OS

TF	p-value
MA0107.1_RELA	0.03
MA0163.1_PLAG1	0.05
MA0492.1_JUND.var.2	0.05
MA0501.1_MAF.NFE2	0.03
MA0046.2_HNF1A	0.007
MA0659.1_MAFG	0.03
MA0711.1_OTX1	0.01
MA0714.1_PITX3	0.01
MA0727.1_NR3C2	0.03
MA0729.1_RARA	0.05
MA0740.1_KLF14	0.04
MA0777.1_MYBL2	0.01
MA0790.1_POU4F1	0.02
MA0791.1_POU4F3	0.008
MA0796.1_TGIF1	0.05
MA0797.1_TGIF2	0.02
MA0833.1_ATF4	0.03
MA0466.2_CEBPB	0.05
MA0838.1_CEBPG	0.05
MA0846.1_FOXC2	0.005
MA0525.2_TP63	0.02
MA0028.2_ELK1	0.03
MA0873.1_HOXD12	0.02
MA0875.1_BARX1	0.006
MA0878.1_CDX1	0.006

C

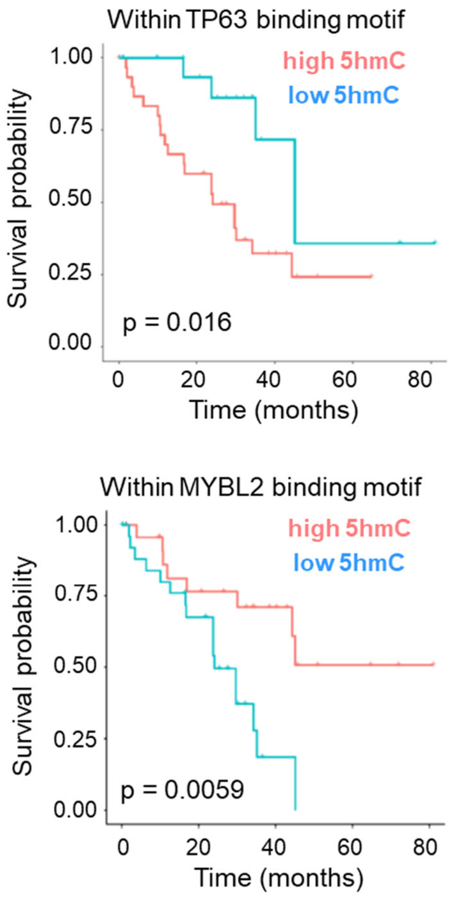

Figure S5. Identification of survival-associated TFs with differential 5hmC enrichment between healthy donors and patients.

(A) Heatmap representation of 5hmC deviation scores at the annotated TF-binding motifs (n = 380) in healthy donors and patients with known TET2 mutation status.

(B) A table listing TFs motifs that showed significant correlations between the differential 5hmC enrichment (healthy donor vs MDS patients) and overall survival (OS) in patients.

(C) Two examples of Kaplan-Meier survival curves of patients with differential 5hmC enrichment within the *TP63* and *MYBL2* binding motifs.

Figure S6

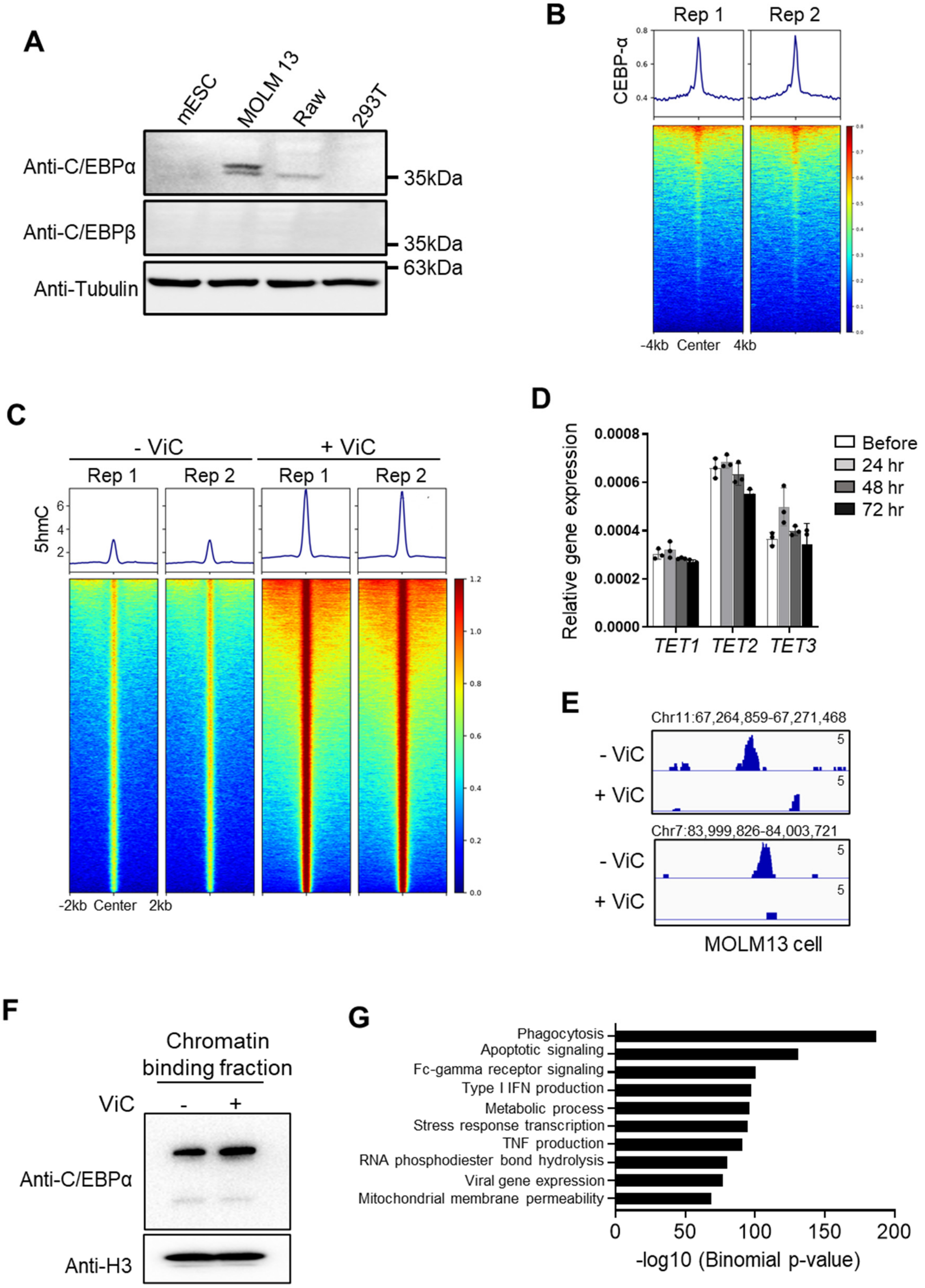

A
mESC
MOLM 13
Raw
293T
Anti-C/EBPα
35kDa
Anti-C/EBPβ
35kDa
63kDa
Anti-Tubulin
B
Rep 1
Rep 2
CEBP-α
-4kb Center 4kb
C
- ViC
+ ViC
Rep 1
Rep 2
Rep 1
Rep 2
5hmC
-2kb Center 2kb
D
Relative gene expression
Before
24 hr
48 hr
72 hr
TET1
TET2
TET3
E
Chr11:67,264,859-67,271,468
- ViC
+ ViC
Chr7:83,999,826-84,003,721
- ViC
+ ViC
MOLM13 cell
F
Chromatin binding fraction
ViC
-
+
Anti-C/EBPα
Anti-H3
G
Phagocytosis
Apoptotic signaling
Fc-gamma receptor signaling
Type I IFN production
Metabolic process
Stress response transcription
TNF production
RNA phosphodiester bond hydrolysis
Viral gene expression
Mitochondrial membrane permeability
-log10 (Binomial p-value)

Figure S6. 5hmC enrichment reshape CEBP-α binding in human leukemia cells

(A) Immunoblot analysis of C/EBP-α and C/EBP-β expression in the indicated cell types. Tubulin was used as control.

(B) Histogram and heatmap representation of C/EBP-α enrichment in MOLM13 cells. A total of 18,959,755 unique reads were identified and 29,748 C/EBP-α peaks were obtained.

(C) Histogram and heatmap representation of 5hmC enrichment in MOLM13 cells with or without vitamin C (ViC) treatment. We identified 39,675 (rep 1) and 44,222 (rep 2) peaks in MOLM13 cells without ViC treatment, and 84,985 (rep 1) and 80,009 (rep 2) in MOLM13 cells treated with ViC, respectively.

(D) Realtime quantitative PCR analysis of *TET1*, *TET2* and *TET3* expression in MOLM13 cells treated with vitamin C at the indicated time points.

(E) Genome-browser views of C/EBP-α enrichment in MOLM13 cells treated with and without vitamin C (ViC, 250 μM) for 72 hrs.

(F) Western-blot analysis of chromatin associated C/EBP-α in MOLM13 cells treated with and without vitamin C (ViC, 250 μM) treatment for 72 hrs.

(F) GREAT analysis of DHMRs identified in MOLM13 cells before and after ViC (250 μM) treatment for 72 hrs.

Figure S7

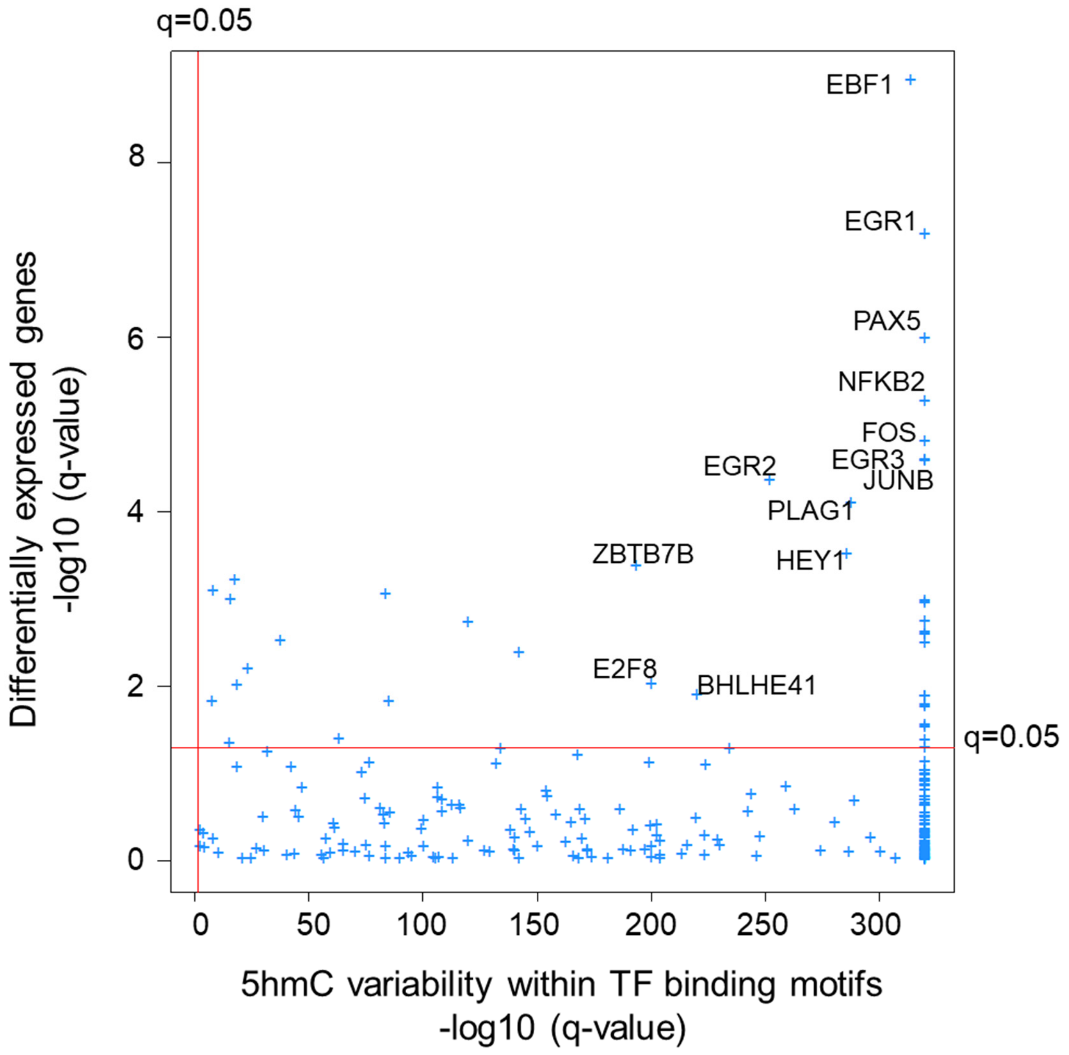

Figure S7. The correlation between 5hmC changes within TF-binding motifs and the expression of TFs in the analyzed cohort.

Scatter-plot represent the changes of 5hmC within the annotated TF-binding motifs (x-axis) and gene expression alterations of the corresponding TFs (y-axis) in patients with myeloid neoplasms compare to healthy donors. The red line represents a q value < 0.05. The TFs that displayed the most dramatic changes in both gene expression and 5hmC levels within their binding motifs were listed in the figure.
